# Author Correction: Insulin-producing organoids engineered from islet and amniotic epithelial cells to treat diabetes

**DOI:** 10.1038/s41467-020-19127-8

**Published:** 2020-10-09

**Authors:** Fanny Lebreton, Vanessa Lavallard, Kevin Bellofatto, Romain Bonnet, Charles H. Wassmer, Lisa Perez, Vakhtang Kalandadze, Antonia Follenzi, Michel Boulvain, Julie Kerr-Conte, David J. Goodman, Domenico Bosco, Thierry Berney, Ekaterine Berishvili

**Affiliations:** 1 Cell Isolation and Transplantation Center, Surgery, Geneva University Hospitals and University of Geneva, Geneva, Switzerland; 2 Institute of Medical Research, Ilia State University, Tbilisi, Georgia; 3 Department of Health Sciences, University of Oriental Piedmont, Novara, Italy; 4 Department of Gynecology and Obstetrics, Geneva University Hospitals and University of Geneva, Geneva, Switzerland; 5 INSERM U1190, Translational Research for Diabetes, University of Lille, Lille, France; 6 Department of Nephrology, St Vincent’s Hospital, Melbourne, VIC Australia

**Keywords:** Regenerative medicine, Tissue engineering

Correction to: *Nature Communications* 10.1038/s41467-019-12472-3, published online 3 October 2019.

In the original version of this Article the ethical registration number permitting collection of amniotic membranes in the Human samples subsection of the “Methods” section was mis-cited as 2017-00101. The correct number refers to the pre-Basec study PB_2017-00101, which is not listed in the Register of Research Projects in Switzerland (RAPS). The old reference number of this study is 14-273 and can be found in a list of protocols submitted before 2015 (https://www.ge.ch/document/liste-protocoles-soumis-2015).

This has been corrected in both the PDF and HTML versions of the Article.

