# Supplementary material for: Insulin-producing organoids engineered from islet and amniotic epithelial cells to treat diabetes
Source: Nat Commun. 2019 Oct 3;10:4491. doi: 10.1038/s41467-019-12472-3 (PMC6776618; doi:10.1038/s41467-019-12472-3)
Supplement: Supplementary file 1 — Supplementary Information [file 41467_2019_12472_MOESM1_ESM.pdf]

Supplementary information

"Insulin-producing organoids engineered from islet- and amniotic epithelial cells to treat diabetes"

Lebreton et al.

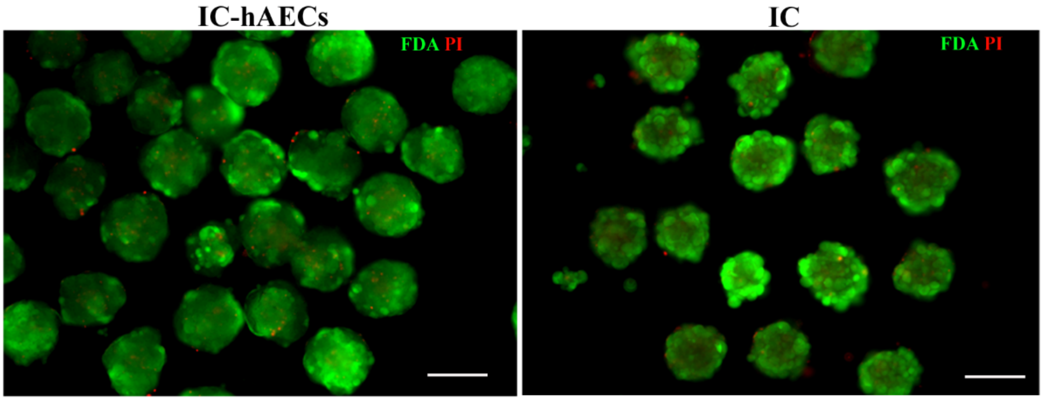

Supplementary Figure 1. Immunofluorescence of IC spheroids and IC-hAEC organoids assessed for viability by FDA/PI test; green (FDI) and red (PI) signals indicate live and dead cells respectively. Scale bars: 100 µm.

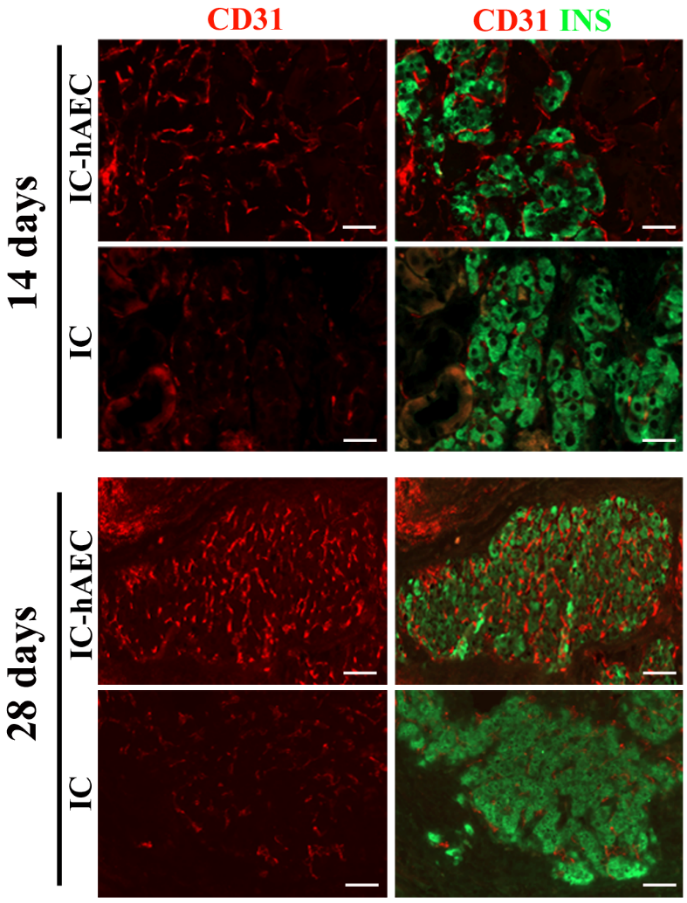

Supplementary Figure 2. Vascular density of the IC-hAEC grafts. IC grafts had noticeably less neovascularization compared to IC-hAEC organoids. The blood vessels of the graft site were detected on days 14 and 28 by CD31 immunostaining (red; n = 10). Beta cells are stained for insulin (green). Scale bars: 20 µm (14 days), 50 µm (28days)

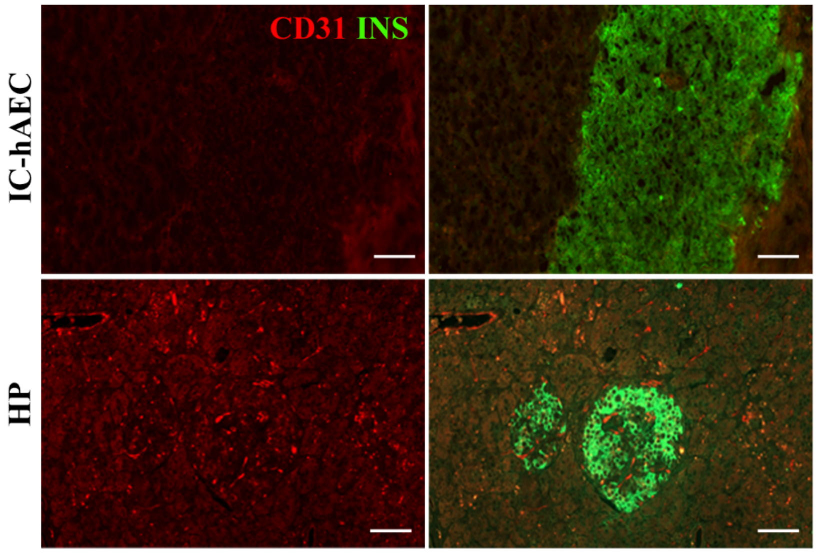

Supplementary Figure 3. Blood vessels were stained with human specific CD31(red) in IC-hAEC grafts and human pancreas (HP). Beta cells are stained for insulin (green). No human derived vessels were detected in the IC-hAEC grafts. Scale bars: 50 µm

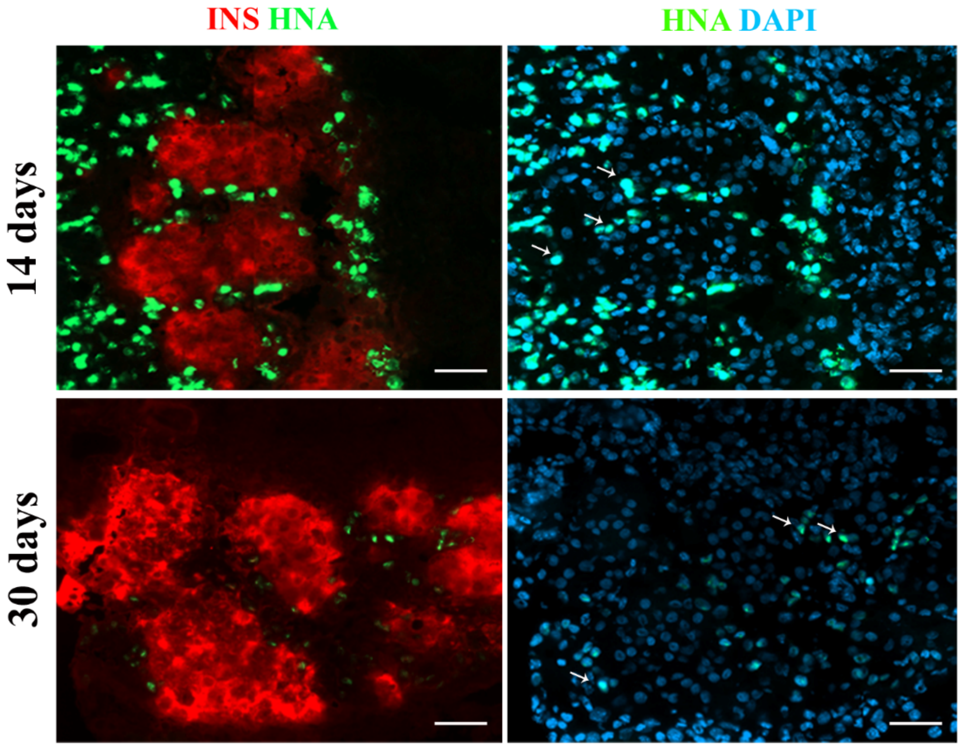

Supplementary Figure 4. hAECs are detectable up to 4 weeks after transplantation. hAECs were visualized at day 14 and 28by using HNA staining (green). Beta cells are stained for insulin (red) and nuclei are stained with DAPI (blue). HNA-DAPI double positive nuclei are indicated by arrows. Scale bars: 20 µm.

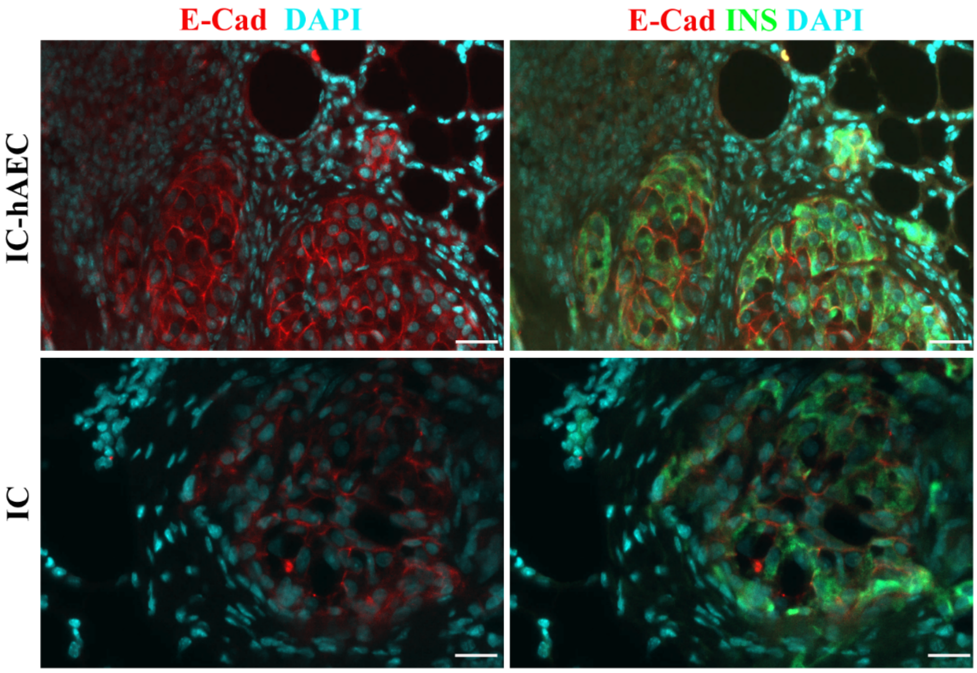

Supplementary Figure 5. Expression of E- cadherin by transplanted human-derived IC spheroids and IC-hAEC organoids into the epididymal fat pad, visualized by immunohistochemistry (red). E cadherin expression was considerably higher in the IC-hAEC group. Beta cells are stained for insulin (green) and nuclei are stained with DAPI (blue). Scale bars: 20 µm

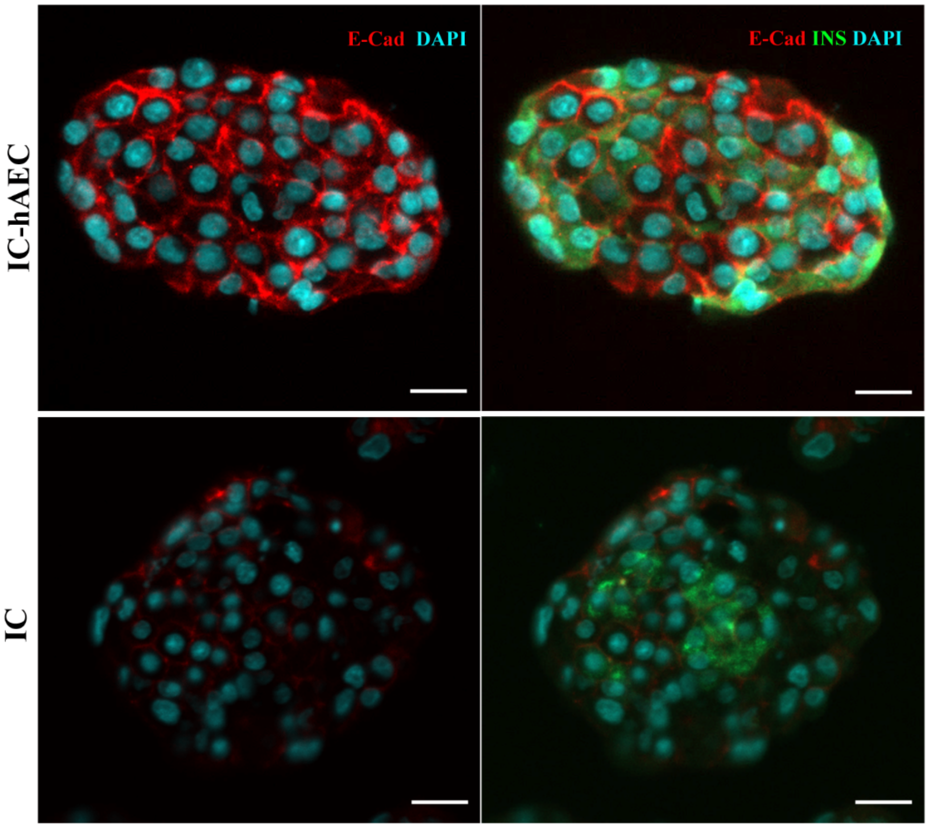

Supplementary Figure 6. In vitro expression of E- cadherin by IC spheroids and IC-hAEC organoids in hypoxic conditions, visualized by immunohistochemistry (red). E cadherin expression was considerably higher in the IC-hAEC group. Beta cells are stained for insulin (green) and nuclei are stained with DAPI (blue). Scale bars: 20 μm

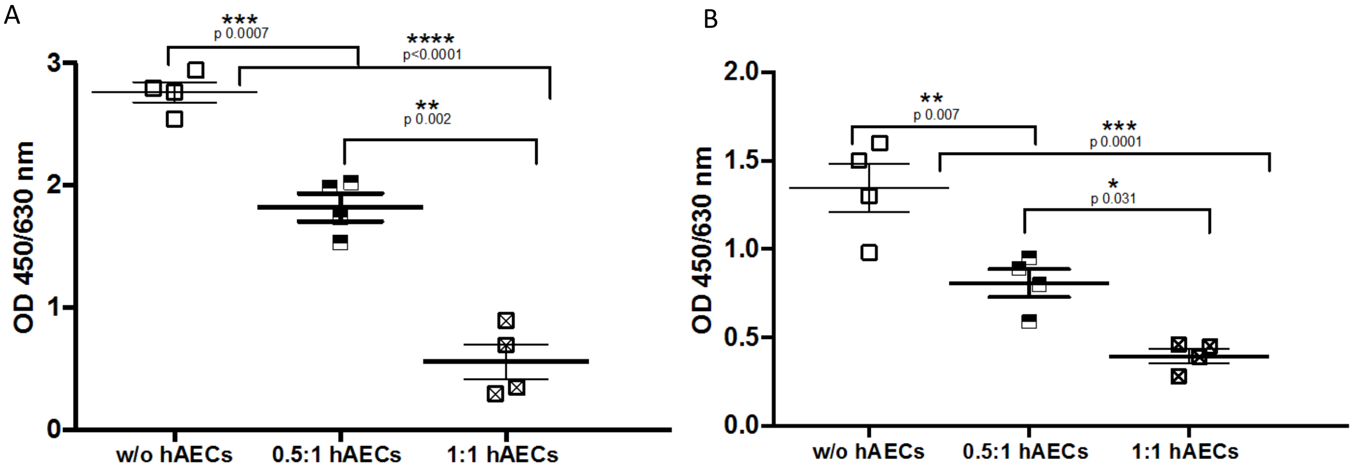

Supplementary Figure 7. hAECs exhibit immunomodulatory potential in MLR cultures. Human PBMCs were stimulated either by allogeneic PBMCs (A) or CD3/CD28 antibodies (B) in the presence or absence of third party hAECs. Lymphocyte proliferation was assessed by Bromo-deoxy-Uridine (BrdU) incorporation in enzyme-linked immunosorbent assays (ELISA). BrdU incorporation by responder PBMCs in the presence of allogeneic cells or mitogens was inhibited by hAECs in a dose-dependent manner. The results are expressed as net absorbance of the stimulated cells minus baseline (absorbance of unstimulated cells). All experiments were repeated with PBMCs from 2 different donors and hAECs derived from four different donors (passage 1). All experiments were done in triplicates. One- way ANOVA with Sidak's multiple comparisons test. Data are shown +/- SEM.

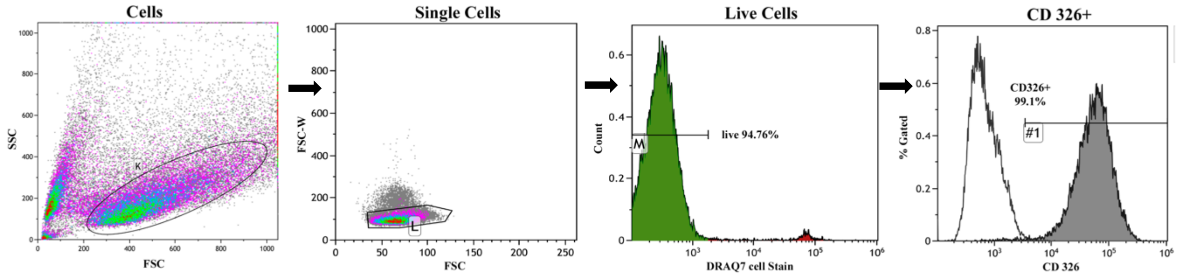

Supplementary Figure 8. Gating strategy used for flow cytometric analysis of single, live hAECs

Supplementary Table 1. Total insulin content (µg/L) in IC spheroids and IC-hAEC organoids in normoxic and hypoxic conditions.

**Normoxia**		**Hypoxia**	
**IC**	**IC-hAECs**	**IC**	**IC-hAECs**
1141.933	1278.02	237.992	709.696
937.063	883.035	246.213	420.055
1289.248	1190.292	396.703	849.778
1563.326	1257.032	105.702	651.66
1365.236	1183.123	232.156	558.356

Supplementary Table 2. List of antibodies used for this study

Primary Antibodies	Company	Country	Product number	Application	Dilution
PerCP-Cy 5.5 Mouse anti-SSEA-4	BD Biosciences	Switzerland	561565	FC	1:50
PE-Cy 7 Mouse anti-human CD90	BD Biosciences	Switzerland	561558	FC	1:100
FITC Mouse anti-human CD105	BD Biosciences	Switzerland	561443	FC	1:50
BV421 Mouse anti-human CD326	BD Biosciences	Switzerland	563180	FC	1:50
PE anti-human HLA-E	Biolegend	UK	342604	FC	1:50
APC anti-human HLA-G	Biolegend	UK	335910	FC	1:50
PE anti-human CD31	Biolegend	UK	303106	FC	1:30
PerCP-Cy 5.5 anti-human CD45	Biolegend	UK	368504	FC	1:25
Purified Mouse IgG1, κ Isotype Ctrl	BD Biosciences	Switzerland	555746	FC	1:50
PerCP-Cy 5.5 Mouse IgG3, κ Isotype Ctrl	BD Biosciences	Switzerland	561572	FC	1:50
PE-Cy 7 Mouse IgG1, κ Isotype Ctrl	BD Biosciences	Switzerland	557872	FC	1:100
FITC Mouse IgG1, κ Isotype Ctrl	BD Biosciences	Switzerland	555748	FC	1:50
BV421 Mouse IgG1, κ Isotype Ctrl	BD Biosciences	Switzerland	562438	FC	1:50
PE Mouse IgG1, κ Isotype Ctrl	Biolegend	UK	400112	FC	1:50
APC Mouse IgG2a, κ Isotype Ctrl	Biolegend	UK	400222	FC	1:50
PerCP-Cy 5.5 Mouse IgG1	Biolegend	UK	400150	FC	1:100
Purified rabbit anti-human CD34	Abcam	UK	ab81289	FC, IF	1:50 (FC) 1:2500 (IF)
Rabbit polyclonal anti wide spectrum Cytokeratin	Abcam	UK	ab9377	IF	1:75
Rabbit polyclonal anti-Oct4	Abcam	UK	ab19857	IF	1:200

Mouse Anti-Ecadherin	Cell Signalling Technology	Maryland (USA)	14472S	IF	1:50
Mouse anti-human nuclear antigen	Lifespan Bosciences	Washington (USA)	LS-C357989	IF	1:200
Rabbit polyclonal anti HIF-1 alpha	Abcam	UK	ab2185	IF	1:100
GuineaPig anti-insulin	DakoCytomation	Switzerland	A0564	IF	1:100
Mouse anti-glucagon	Sigma Aldrich	Switzerland	G2654	IF	1:4000
Rabbit anti-Somatostatin	DakoCytomation	Switzerland	A0566	IF	1:100
Rabbit anti mouse CD31	Abcam	UK	Ab28364	IF	1:50
Mouse anti-human CD31	DakoCytomation	Switzerland	M0823	IF	1:50
Mouse Anti-E-cadherin	Cell Signalling Technology	Maryland (USA)	14472S	IF	1:50
rabbit anti-human VEGF	Abcam	UK	Ab52917	IF	1:200
Rabbit anti-Laminin	Sigma Aldrich	Switzerland	L9393	IF	1:30
Rabbit anti Human Collagen IV	Bio-Rad	Switzerland	2150-0140	IF	1:30
**Secondary Antibodies**	**Company**	**Country**	**Product number**	**Application**	**Dilution**
AlexaFluor 488 goat anti-mouse IgG	ThermoFisher Scientific	Switzerland	A11001	IF	1:300
AlexaFluor 488 goat anti-rabbit IgG	ThermoFisher Scientific	Switzerland	A11008	IF	1:300
AlexaFluor 555 donkey anti-rabbit IgG	ThermoFisher Scientific	Switzerland	A31572	IF	1:300
AlexaFluor 555 donkey anti-mouse IgG	ThermoFisher Scientific	Switzerland	A31570	IF	1:300
Goat anti GuineaPig Fluorescein IgG	Jackson Laboratories	Switzerland	106-095-003	IF	1:200
FITC Goat Anti-Mouse IgG/IgM	BD Biosciences	Switzerland	555988	FC	1:100

Supplementary Table 3. List of ELISA kits used for this study

Product	Company	Country	Product number
Rat insulin ELISA kit	Mercodia	Sweden	10-1250-01
Insulin ELISA kit	Mercodia	Sweden	10-1113-01
rat C-peptide ELISA	Mercodia	Sweden	10-1172-01
Ultrasensitive C-peptide ELISA	Mercodia	Sweden	10-1141-01

Supplementary Table 4. RT qPCR reagents

Product	Company	Country	Product number
Rneasy minikit	Qiagen	France	74104
High Capacity cDNA Reverse transcription kit	ThermoFisher Scientific	Switzerland	4368814
TaqMan Fast Advance Master Mix	ThermoFisher Scientific	Switzerland	4444557
Takyon No-Rox SYBR Core Kit blue dTTP	Takyon	Belgium	UF-NSCT-B0201

Gene	Forward Primer	Reverse Primer	Amplification method
Rat ACTB	Rn00667869-m1		Taqman
Rat INS2	Rn01774648-g1		Taqman
Rat BCL2	Rn99999125-m1		Taqman
Rat CASP3	Rn00563902-m1		Taqman
Rat CASP8	Rn00574069-m1		Taqman
Rat CASP9	Rn00581212-m1		Taqman
Rat RPLP1	TCT CTG AGC TTG CCT GCA TCT ACT	CCT ACA TTG CAG ATG AGG CTT CCA	SYBR
Rat VEGF	AAC GCG AGT CTG TGT TTT TGC	AGC GGA GAA AGC ATT TGT TTG	SYBR

Supplementary Table 5. Media and reagents

Product	Company	Country	Product number
DMEM/F-12	ThermoFisher Scientific	Switzerland	21041-25
MEM NEAA 100X	ThermoFisher Scientific	Switzerland	11140-035
HBSS	ThermoFisher Scientific	Switzerland	14175-053
Dulbecco's Phosphate Buffer Saline (dPBS)	Sigma Aldrich	Switzerland	D8537
Fetal bovine serum (FBS)	Merk Millipore	Switzerland	s0115
Bovine Serum Albumine (BSA)	Sigma Aldrich	Switzerland	A3733
human recombinant epidermal growth factor (hEGF)	Sigma Aldrich	Switzerland	E9644
Sodium Pyruvate	Sigma Aldrich	Switzerland	s8636
L-Glutamin-Penicillin-Streptomycin 10X	Sigma Aldrich	Switzerland	G1146
Fungin	Invivogen	USA	ant-fn-2
2-mercaptoethanol	ThermoFisher Scientific	Switzerland	21985-023
collagenase V	Sigma Aldrich	Switzerland	C9263-5G
StemPro® Accutase®	ThermoFisher Scientific	Switzerland	A11105-01
0.05% (w/v) trypsin-EDTA	ThermoFisher Scientific	Switzerland	25300-054
CM-DiI	ThermoFisher Scientific	Switzerland	C7000
CM-DiO	ThermoFisher Scientific	Switzerland	V22886
Draq7	Biostatus	Shepshed UK	DR71000
