# Supplementary material for: Insulin-producing organoids engineered from islet and amniotic epithelial cells to treat diabetes
Source: Nat Commun. 2019 Oct 3;10:4491. doi: 10.1038/s41467-019-12472-3 (PMC6776618; doi:10.1038/s41467-019-12472-3)
Supplement: Supplementary file 3 — Reporting Summary [file 41467_2019_12472_MOESM3_ESM.pdf]

nature research

Corresponding author(s): Ekaterine Bershvili

Last updated by author(s): Sep 10, 2019

Reporting Summary

Nature Research wishes to improve the reproducibility of the work that we publish. This form provides structure for consistency and transparency in reporting. For further information on Nature Research policies, see Authors & Referees and the Editorial Policy Checklist.

Statistics

For all statistical analyses, confirm that the following items are present in the figure legend, table legend, main text, or Methods section.

n/a	Confirmed	
☐	☒	The exact sample size (*n*) for each experimental group/condition, given as a discrete number and unit of measurement
☐	☒	A statement on whether measurements were taken from distinct samples or whether the same sample was measured repeatedly
☐	☒	The statistical test(s) used AND whether they are one- or two-sided<br>*Only common tests should be described solely by name; describe more complex techniques in the Methods section.*
☒	☐	A description of all covariates tested
☒	☐	A description of any assumptions or corrections, such as tests of normality and adjustment for multiple comparisons
☐	☒	A full description of the statistical parameters including central tendency (e.g. means) or other basic estimates (e.g. regression coefficient) AND variation (e.g. standard deviation) or associated estimates of uncertainty (e.g. confidence intervals)
☒	☐	For null hypothesis testing, the test statistic (e.g. *F*, *t*, *r*) with confidence intervals, effect sizes, degrees of freedom and *P* value noted<br>*Give P values as exact values whenever suitable.*
☒	☐	For Bayesian analysis, information on the choice of priors and Markov chain Monte Carlo settings
☒	☐	For hierarchical and complex designs, identification of the appropriate level for tests and full reporting of outcomes
☒	☐	Estimates of effect sizes (e.g. Cohen's *d*, Pearson's *r*), indicating how they were calculated

Our web collection on statistics for biologists contains articles on many of the points above.

Software and code

Policy information about availability of computer code

Data collection	Information about data collection is provided in the material and methods section of the manuscript
Data analysis	Cells were analyzed by flow cytometry using Kaluza Analysis software from Beckman Coulter (Version 1.5.20365.16139). Morphometry and fluorescent analysis were performed using ImageJ software. All statistical analyses were performed with Prism software 7.02 (GraphPad, La Jolla, CA, USA) or Microsoft Excel. All information about software is reported in the material and methods section of the manuscript.

For manuscripts utilizing custom algorithms or software that are central to the research but not yet described in published literature, software must be made available to editors/reviewers. We strongly encourage code deposition in a community repository (e.g. GitHub). See the Nature Research guidelines for submitting code & software for further information.

Data

Policy information about availability of data

All manuscripts must include a data availability statement. This statement should provide the following information, where applicable:

- Accession codes, unique identifiers, or web links for publicly available datasets
- A list of figures that have associated raw data
- A description of any restrictions on data availability

The source data underlying the main Figs. 1b, 2c, e, 3b, c, e, f, 4a, c, e, f, 5b-c, 6b-d, 7b-c, e-f, h, 8a-b and 9b are provided as a Source Data file.

Field-specific reporting

Please select the one below that is the best fit for your research. If you are not sure, read the appropriate sections before making your selection.

☒ Life sciences ☐ Behavioural & social sciences ☐ Ecological, evolutionary & environmental sciences

For a reference copy of the document with all sections, see nature.com/documents/nr-reporting-summary-flat.pdf

Life sciences study design

All studies must disclose on these points even when the disclosure is negative.

Sample size	Sample size for each experiment is indicated in the corresponding figure legend. Sample size for experiments involving mice receiving rat derived organoids was always determined to be adequate based on the magnitude and consistency of measurable differences between groups.<br>However, sample numbers in the transplantation experiments to cure diabetic mice with human derived organoids were limited by the availability of the human donor samples in the limited time-window. We performed 2 independent experiments using total 2 donors' samples.
Data exclusions	In STZ- induced diabetic mouse models, only mice with blood glucose levels >20 mmol/l for at least 3 consecutive days were used.
Replication	All the data presented in this study were reproduced, so that our data are based on at least three biologically independent experiments with similar results.
Randomization	For all animal experiments, mice were randomly allocated into each experimental group.
Blinding	Investigators performing tissue collection and analysis were blind to group allocation.

Reporting for specific materials, systems and methods

We require information from authors about some types of materials, experimental systems and methods used in many studies. Here, indicate whether each material, system or method listed is relevant to your study. If you are not sure if a list item applies to your research, read the appropriate section before selecting a response.

Materials & experimental systems

n/a	Involved in the study	
☐	☒	Antibodies
☒	☐	Eukaryotic cell lines
☒	☐	Palaeontology
☐	☒	Animals and other organisms
☐	☒	Human research participants
☒	☐	Clinical data

Methods

n/a	Involved in the study	
☒	☐	ChIP-seq
☐	☒	Flow cytometry
☒	☐	MRI-based neuroimaging

Antibodies

Antibodies used	All antibodies for flow cytometry and immunofluorescence are stated in the Methods sections and listed in supplementary Table 2. Supplier, catalogue number and dilution used are included.
Validation	Only commercially available antibodies were used. Validated was performed as per manufacturer's instructions.

Animals and other organisms

Policy information about studies involving animals; ARRIVE guidelines recommended for reporting animal research

Laboratory animals	Male SCID mice and male Sprague-Dawley (SD) rats were obtained from Janvier Labs (Le Genest St-Isle, France).
Wild animals	N/A
Field-collected samples	N/A
Ethics oversight	All animal procedures were performed according to protocols approved by the University of Geneva Institutional Animal Care and Use Committee.

Note that full information on the approval of the study protocol must also be provided in the manuscript.

Human research participants

Policy information about studies involving human research participants

Population characteristics	Amniotic membranes were obtained from term healthy placentas of women undergoing elective cesarean section, under CCER protocol 2017-00101. Human islets isolated from brain-dead multiorgan donors were obtained from the Lille University Hospital. The use of human islets for research was approved by CCER protocol 2016-01979.
Recruitment	Placentas were donated by women undergoing delivery by elective cesarean section, after having obtained Informed, written consent prior to amniotic tissue collection. Islets were obtained from brain-dead multiorgan donors after obtaining consent to donation from the next of kin.
Ethics oversight	Studies involving human tissues were approved by the Commission Cantonale d'Ethique de la Recherche (CCER), in compliance with the Swiss Human Research Act (810.30).

Note that full information on the approval of the study protocol must also be provided in the manuscript.

Flow Cytometry

Plots

Confirm that:

- [x] The axis labels state the marker and fluorochrome used (e.g. CD4-FITC).
- [x] The axis scales are clearly visible. Include numbers along axes only for bottom left plot of group (a 'group' is an analysis of identical markers).
- [x] All plots are contour plots with outliers or pseudocolor plots.
- [x] A numerical value for number of cells or percentage (with statistics) is provided.

Methodology

Sample preparation	Sample preparation is described in the method section of the manuscript.
Instrument	Gallios cytometer (Beckman Coulter, Indianapolis, IN, USA)
Software	Kaluza Analysis software from Beckman Coulter (Version 1.5.20365.16139).
Cell population abundance	Cell sorting was not performed
Gating strategy	Samples were first gated to eliminate debris (by FSC vs SSC), then gated on single cells (FSC-W vs FSC-H) following dead cell exclusion (as described in the method section) and analysis for the expression of specific markers. Gating strategy is shown in Supplementary Figure 7 with CD326 staining as an example.

- [x] Tick this box to confirm that a figure exemplifying the gating strategy is provided in the Supplementary Information.
